# Umbilical Cord pH Levels and Neonatal Morbidity and Mortality

**DOI:** 10.1001/jamanetworkopen.2024.27604

**Published:** 2024-08-14

**Authors:** Charlotte Brix Andersson, Claus Klingenberg, Line Thellesen, Søren Paaske Johnsen, Ulrik Schiøler Kesmodel, Jesper Padkaer Petersen

**Affiliations:** 1 Danish Center for Health Services Research, Department of Clinical Medicine, Aalborg University, Aalborg, Denmark; 2 Department of Obstetrics and Gynaecology, Aalborg University Hospital, Thisted, Denmark; 3 Department of Paediatrics, University Hospital of North Norway, Tromsø; 4 Research Group for Child and Adolescent Health, Department of Clinical Medicine, The Arctic University of Norway, Tromsø; 5 Department of Obstetrics and Gynaecology, Herlev Hospital, University of Copenhagen, Herlev, Denmark; 6 Department of Obstetrics and Gynaecology, Aalborg University Hospital, Aalborg, Denmark; 7 Department of Clinical Medicine, Aalborg University, Aalborg, Denmark; 8 Department of Paediatrics, Aarhus University Hospital, Aarhus, Denmark

## Abstract

**Importance:**

Umbilical cord pH (UC-pH) level is an important objective indicator of intrapartum fetal hypoxia and is used to predict neonatal morbidity and mortality. A UC-pH value of less than 7.00 is often defined as a threshold for severe acidosis, but existing evidence is divergent and largely based on UC-pH measurements from selected populations; consequently, the results are challenging to interpret.

**Objective:**

To investigate the association between UC-pH levels and the risk of adverse neonatal outcomes in a national setting with universal UC-pH measurement.

**Design, Setting, and Participants:**

This national, population-based cohort study included all liveborn, singleton, full-term infants without malformations born in Denmark from January 1, 2012, to December 31, 2018. Data were analyzed from January 1, 2023, to March 1, 2024.

**Exposure:**

Umbilical cord pH level categorized as less than 7.00, 7.00 to 7.09, 7.10 to 7.19 and 7.20 to 7.50 (reference group).

**Main Outcomes and Measures:**

The primary outcome was a composite of severe adverse neonatal outcomes: neonatal death, therapeutic hypothermia, mechanical ventilation, treatment with inhaled nitric oxide, or seizures. Secondary outcomes were individual components of the primary outcome, Apgar score, respiratory outcomes, and hypoglycemia. Data are presented as adjusted risk ratios (ARRs) with 95% CIs.

**Results:**

Among the 340 431 infants included, mean (SD) gestational age was 39.9 (1.6) weeks; mean (SD) birth weight was 3561 (480) g; and 51.3% were male. Umbilical cord pH of less than 7.20 was observed more often among infants with a gestational age of 40 or 41 weeks (31.6%-33.6% compared with 18.2%-20.2% at a gestational age of 39 weeks) and among male infants (53.9%-55.4% vs 44.6%-46.1% among female infants). Compared with the pH reference group (576 of 253 540 [0.2%]), the risk for the primary outcome was increased for the groups with UC-pH levels of less than 7.00 (171 of 1743 [9.8%]), 7.00 to 7.09 (101 of 11 904 [0.8%]), and 7.10 to 7.19 (259 of 73 244 [0.4%]). Comparable patterns were observed for the individual outcomes, except for neonatal death, which was only increased in the group with UC-pH levels of less than 7.10. The risk of treatment with continuous positive airway pressure was increased when UC-pH levels were less than 7.20, and the risk of hypoglycemia was 21.5% if UC-pH levels were less than 7.10.

**Conclusions and Relevance:**

In this cohort study of 340 431 newborn infants, results support and extend previous studies indicating a higher risk of adverse outcomes even at UC-pH levels above 7.00. The threshold for more intensive observation and treatment may be reconsidered.

## Key Points

**Question:**

What is the umbilical cord (UC) pH threshold for increased morbidity and mortality among full-term infants in a national unselected population?

**Findings:**

In this national cohort study of 340 431 liveborn, singleton, full-term infants without malformations, lower UC-pH levels were associated with the risk of adverse neonatal outcomes, even at levels not previously associated with increased morbidity and mortality. This association was observed for serious and less severe outcomes, including the need for respiratory support during the transition to extrauterine life and hypoglycemia.

**Meaning:**

These findings suggest that the UC-pH threshold for more intensive observation and treatment, in case of acidosis, may be reconsidered.

## Teaser

This cohort study investigates the association between umbilical cord pH values and the risk of adverse neonatal outcomes among newborns in Denmark, which has universal umbilical cord pH measurement.

## Introduction

Umbilical cord blood pH (UC-pH) measurement is used to identify infants exposed to intrapartum hypoxia and acidosis. Severe metabolic acidosis is associated with an increased risk of developing hypoxic-ischemic encephalopathy (HIE), potentially leading to lifelong disabilities or death.^1,2^ Additionally, severe intrapartum hypoxia can result in multiorgan injury, affecting the heart, lungs, and kidneys.^3^ Immediate recognition of severe acidosis after birth is essential for closer observation and timely intervention, and in case of HIE, to provide therapeutic hypothermia aimed at preventing the second phase of brain injury that occurs 6 to 15 hours after birth.^4,5,6^

There is no global consensus on a specific UC-pH threshold value associated with increased morbidity and mortality risk. A UC-pH level of less than 7.00 is often defined as severe acidosis, and this threshold is also widely used as one of the criteria for therapeutic hypothermia.^4,5,6^ Some authors argue that a higher UC-pH threshold improves detection of infants with moderate to severe HIE who may benefit from therapeutic hypothermia, and many centers today already screen for HIE and offer therapeutic hypothermia to infants with UC-pH values above 7.00 if other criteria are fulfilled, but evidence of benefit is limited.^7,8,9,10^ For respiratory adverse outcomes, previous studies^11,12^ have shown that need for respiratory support to assist transition to extrauterine life is increased at values above 7.00. In 2022, Bligard et al^11^ investigated adverse neonatal outcomes among 2081 infants born from planned cesarean section with regional anesthesia and found an almost 3-fold increased risk of respiratory distress among infants with UC-pH levels of less than 7.20 compared with infants with UC-pH levels of 7.20 or greater.

Most previous studies are based on populations in settings with a policy of selective measurement of UC-pH levels, reserved for high-risk deliveries or deliveries in which the fetus shows sign of hypoxia during birth. Since 2009, the policy in Denmark has been universal measuring of UC-pH levels, and in 2014 the proportion of infants with at least 1 UC-pH measurement was 95.6%. The implementation of universal UC-pH measurement has increased the proportion of samples in both vigorous and nonvigorous infants and in acute obstetric situations.^13^ In this population-based cohort study, we investigated the association between UC-pH values and the risk of adverse neonatal outcomes in a setting with universal UC-pH measurement.

## Methods

Permission to access data used for the study was granted by the Danish Data Protection Agency. In Denmark, ethical permissions and informed consent are not required for register-based studies. The study was reported according to the Strengthening the Reporting of Observational Studies in Epidemiology (STROBE) reporting guideline.

### Study Design and Setting

In this population-based cohort study, data were obtained from the Danish Civil Registration System, which holds information on residence and vital status, and the Danish National Patient Register. The Danish National Patient Register contains data on all pregnant women, deliveries, and newborn infants in Denmark, including obstetric characteristics, complications, UC-pH levels, diagnoses (*International Statistical Classification of Diseases, Tenth Revision*) and medical procedural codes.^14,15^ The unique 10-digit civil registration number, assigned to all inhabitants of Denmark at birth or entry into the country, allows unambiguous individual-level identification and data linkage across nationwide registers, including linkage between mother and infant.

In Denmark, measurement of UC-pH levels is recommended from blood samples collected within the first minute after birth in case the cord is not clamped, and within 30 minutes after birth if from a clamped segment of the cord. It is recommended that the blood gas levels be analyzed within 30 minutes from birth.^16^ Late cord clamping was recommended in Denmark from 2017 and was not common practice during the study period.

### Study Population

This study was based on a secondary data analysis of all liveborn, singleton, full-term infants (gestational age ≥37 weeks) born in Denmark from January 1, 2012, through December 31, 2018. Infants with congenital malformations of the heart, lungs, or nervous system were excluded. Furthermore, we excluded infants with UC-pH levels of less than 6.50 and greater than 7.50, as these pH values were considered registration errors (Figure).^17^

**Figure.**
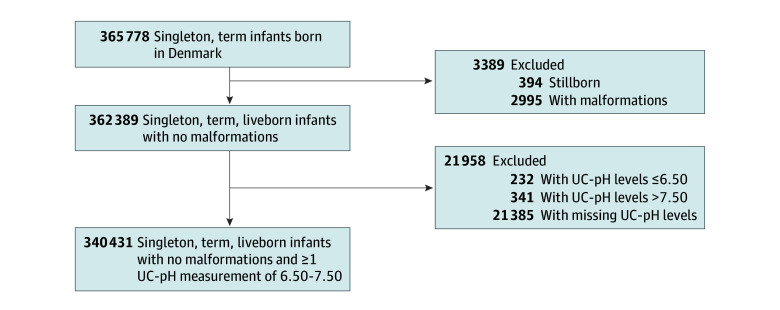
Study Population UC-pH indicates umbilical cord pH.

We included all infants with at least 1 UC-pH measurement. Infants were categorized into groups according to UC-pH levels of less than 7.00, 7.00 to 7.09, 7.10 to 7.19, and 7.20 to 7.50 (reference group). The reference group was chosen since this interval contains the top of the normal reference curve.^10,17^

### Clinical Information and Definitions

We obtained data on gestational age, sex assigned at birth, birth weight, maternal smoking in pregnancy, types 1 and 2 diabetes, gestational diabetes, hypertension, preeclampsia, other medical diseases (respiratory diseases, hypothyroidism, hyperthyroidism, polycystic ovary syndrome, gastrointestinal diseases, and neurological disease), placental insufficiency (pathological signs on the cardiotocography, intrauterine growth restriction [<−2 SD], low amniotic fluid volume, or abnormal ultrasonographic Doppler indices of fetal vessels), intrapartum fever, serious birth events (shoulder dystocia, uterine rupture, placental abruption, cord prolapse, or vasa praevia), breech presentation, instrumental delivery, and emergency cesarean section (Table 1). We did not report data on race and ethnicity since we did not find it relevant in this context. Gestational age was set in 99.2% of pregnancies during an ultrasonographic examination early or late in the second trimester.^18^ Diagnoses and procedures included in this study are presented in eTable 1 in Supplement 1).

**Table 1.** Baseline Descriptive Characteristics According to UC-pH

Characteristic	UC-pH group^a^			
	<7.00 (n = 1743)	7.00-7.09 (n = 11 904)	7.10-7.19 (n = 73 244)	7.20-7.50 (n = 253 540)
Infant				
Gestational age, mean (SD), wk	40.2 (1.2)	40.2 (1.2)	40.1 (1.1)	39.9 (1.2)
Gestational age, wk				
37	82 (4.7)	430 (3.6)	2727 (3.7)	12 648 (5.0)
38	162 (9.3)	1071 (9.0)	7496 (10.2)	37 951 (15.0)
39	318 (18.2)	2229 (18.7)	14 819 (20.2)	61 688 (24.3)
40	568 (32.6)	3763 (31.6)	24 028 (32.8)	76 299 (30.1)
41	555 (31.8)	4000 (33.6)	22 041 (30.1)	60 045 (23.7)
≥42	58 (3.3)	411 (3.5)	2133 (2.9)	4909 (1.9)
Sex assigned at birth				
Male	966 (55.4)	6522 (54.8)	39 468 (53.9)	127 602 (50.3)
Female	777 (44.6)	5382 (45.2)	33 776 (46.1)	125 938 (49.7)
Birth weight, mean (SD), g^b^	3552 (537)	3607 (491)	3615 (477)	3545 (478)
SGA <1 SD	265 (15.2)	1469 (12.3)	7645 (10.4)	27 525 (10.9)
SGA <2 SD	78 (4.5)	1469 (12.3)	2200 (3.0)	27 525 (10.9)
LGA >2 SD	150 (8.6)	1216 (10.2)	7729 (10.6)	24 267 (9.6)
Pregnancy				
Smoking in pregnancy	117 (6.7)	668 (5.6)	4621 (6.3)	18 796 (7.4)
Type 1 or 2 diabetes	15 (0.9)	115 (1.0)	659 (0.9)	1932 (0.8)
Gestational diabetes	75 (4.3)	492 (4.1)	2960 (4.0)	9354 (3.7)
Hypertension	49 (2.8)	388 (3.3)	2208 (3.0)	6805 (2.7)
Other medical disease^c^	239 (13.7)	1179 (9.9)	7167 (9.8)	24 342 (9.6)
Placental insufficiency^d^	126 (7.2)	544 (4.6)	3394 (4.6)	14 212 (5.6)
Birth				
Intrapartum fever	64 (3.7)	578 (4.9)	2915 (4.0)	6275 (2.5)
Serious birth events^e^	159 (9.1)	437 (3.7)	1724 (2.4)	3702 (1.5)
Breech, vaginal delivery	66 (3.8)	193 (1.6)	882 (1.2)	9038 (3.6)
Instrumental delivery	500 (28.7)	2708 (22.7)	9815 (13.4)	12 113 (4.8)
Emergency cesarean section	478 (27.4)	1189 (10.0)	4531 (6.2)	26 814 (10.6)

Abbreviations: LGA, large for gestational age; SGA, small for gestational age; UC-pH, umbilical cord pH.

^a^ Data are presented as No. (%) of infants unless otherwise indicated. Percentages have been rounded and may not total 100.

^b^ Birth weight data were not available or not valid for 2575 infants.

^c^ Includes respiratory diseases, hypothyroidism, hyperthyroidism, polycystic ovary syndrome, gastrointestinal tract diseases, neurological disease, anemia, and intrahepatic cholestasis of pregnancy.

^d^ Includes signs of fetal distress before labor, pathological signs on the cardiotocography, intrauterine growth restriction (≤2 SD), low amnion fluid volumen, or abnormal ultrasonographic Doppler indexes of fetal vessels.

^e^ Includes shoulder dystocia, uterine rupture, abruptio placentae, cord prolapse, or vasa praevia.

The outcomes of interest were neonatal morbidity and mortality, occurring within the first 28 days after birth. The primary outcome was defined as a composite of severe adverse neonatal outcomes encompassing neonatal death, therapeutic hypothermia, mechanical ventilation, treatment with inhaled nitric oxide, or seizures. Secondary outcomes were individual components of the primary outcome and specific clinical outcomes, including 5-minute Apgar scores below 4 and 7, treatment with continuous positive airway pressure, meconium aspiration syndrome, and hypoglycemia.

### Statistical Analysis

#### Main Analysis

Data were analyzed from January 1, 2023, to March 1, 2024. Results in the main analysis were adjusted for factors known from the literature to affect the association between hypoxia and adverse neonatal outcomes: infant sex assigned at birth; gestational age (37, 38, 39, 40, 41, and ≥42 weeks), year of birth, intrapartum fever, intrauterine growth restriction, and types 1 and 2 diabetes.^19,20,21,22,23^ Our results are based on 1 UC-pH value from each included infant, and if 2 values were registered, we used the lowest value.

All outcomes, including the composite outcome, were dichotomous and coded as 0 or 1. We used modified Poisson regression models (Poisson regression with robust error variance) to estimate the difference in adverse outcomes between the groups with UC-pH levels below 7.20 and the reference group with UC-pH levels of 7.20 to 7.50.^24,25,26,27^ The results are reported as risks and adjusted risk ratios (ARR) with 95% CIs. Stata, version 15 (StataCorp LLC) was used for statistical analyses.

#### Sensitivity Analysis

We performed 4 sensitivity analyses: one with more restricted composite outcomes, one with a restricted surveillance period of 7 days, one without adjustments, and one only including infants with UC-pH values sampled from both the umbilical artery and vein. Moreover, we analyzed the group with missing UC-pH measurement according to characteristics and outcomes and report on results after multivariate imputation by chained equations for missing UC-pH measurement.^28^

## Results

### Patient Characteristics

Among 362 389 live singleton infants with no malformations in Denmark from 2012 to 2018, at least 1 UC-pH measurement was registered in 340 431 infants (93.9%) (Figure). A total of 5.9% of infants were missing UC-pH measurements, which was higher among infants with the primary outcome (109 of 1216 [9.0%]) and with neonatal death (27 of 141 [19.1%]). The mean (SD) gestational age was 39.9 (1.6) weeks, mean (SD) birth weight was 3561 (480) g, 51.3% were male, and 48.7% were female. Comparison groups included 1743 infants (0.5%) with UC-pH levels of less than 7.00, 11 904 (3.5%) with levels of 7.00 to 7.09, and 73 244 (21.5%) with levels of 7.10 to 7.19. Umbilical cord pH levels of less than 7.20 were observed more often among infants with a gestational age of 40 weeks (568 of 1743 [32.6%] for <7.00; 3763 of 11 904 [31.6%] for 7.00-7.09; 24 028 of 73 244 [32.8%] for 7.10-7.19) and among infants with a gestational age 41 weeks (555 of 1743 [31.8%] for <7.00; 4000 of 11 904 [33.6%] for 7.00-7.09; 22 041 of 73 244 [30.1%] for 7.10-7.19) compared with a gestational age of 39 weeks (318 of 1743 [18.2%] for <7.00; 2229 of 11 904 [18.7%] for 7.00-7.09; 14 819 of 73 244 [20.2%] for 7.10-7.19]) and among male infants (966 [55.4%] for <7.00; 6522 of 11 904 [54.8%] for 7.00-7.09; 39 468 of 73 244 [53.9%] for 7.10-7.19) compared with female infants (777 of 1797 [43.2%] for <7.00; 5382 of 11 904 [45.2%] for 7.00-7.09; 33 776 of 73 244 [46.1%] for 7.10-7.19).

Among infants with UC-pH levels of less than 7.00, there was a higher proportion of pregnancies complicated by placental insufficiency, gestational diabetes, hypertensive disorders, and other maternal medical diseases. Moreover, deliveries were more often complicated by serious birth events (Table 1).

### Adverse Neonatal Outcomes

In the group with UC-pH levels of less than 7.00, the risk of the primary composite outcome was markedly increased compared with the risk in the reference group (171 of 1743 [9.8%] vs 576 of 253 540 [0.2%]), as was the risk of neonatal death (34 of 1743 [2.0%] vs 55 of 253 540 [0.02%]). Comparable differences were seen for all individual outcomes.

In the group with UC-pH levels of 7.00 to 7.09, the risk of the primary outcome was 101 of 11 904 (0.8%). The risk of most adverse outcomes was markedly lower compared with those with UC-pH levels of less than 7.00, but still significantly higher than in the reference group. The ARR for therapeutic hypothermia was 13.83 (95% CI, 8.43-22.69) in the group with UC-pH levels of 7.00 to 7.09 compared with the reference group. The proportion of infants diagnosed with hypoglycemia was comparable if UC-pH levels were less than 7.00 (274 of 1743 [21.5%]) and 7.00 to 7.09 (2449 of 11 904 [20.6%]).

In the group with UC-pH levels of 7.10 to 7.19, the risk of most adverse neonatal outcomes was lower but still increased (primary outcome, 259 of 73 244 [0.4%]). The ARR for the primary outcome was 1.60 (95% CI, 1.38-1.85) and ARRs were 2.24 (95% CI, 20.1-2.50) for low Apgar scores, 1.97 (95% CI. 1.90-2.05) for continuous positive air pressure, and 1.76 (95% CI, 1.70-1.82) for hypoglycemia when compared with infants with UC-pH levels of 7.20 to 7.50. However, the risk of neonatal death was not increased. Overall, 1976 of 11 904 infants (16.6%) with UC-pH levels of 7.00 to 7.09 and 4286 of 73 244 (5.9%) with UC-pH levels of 7.10 to 7.19 received noninvasive respiratory support to assist transition to extrauterine life (ARRs, 5.55 [95% CI 5.30-5.82] and 1.97 [95% CI 1.90-2.05], respectively) (Table 2).

**Table 2.** Comparison of Primary and Secondary Outcomes Between UC-pH Groups

Outcome	UC-pH group, No. (%)				ARR (95% CI)^a^		
	<7.00 (n = 1743)	7.00-7.09 (n = 11 904)	7.10-7.19 (n = 73 244)	7.20-7.50 (n = 253 540)	<7.00	7.00-7.09	7.10-7.19
Primary composite^b^	171 (9.8)	101 (0.8)	259 (0.4)	576 (0.2)	43.93 (37.19-51.90)	3.83 (3.10-4.74)	1.60 (1.38-1.85)
Secondary							
Components of the composite							
Neonatal death	34 (2.0)	11 (0.1)	14 (0.02)	55 (0.02)	93.74 (60.48->100)	4.59 (2.38-8.85)	0.95 (0.53-1.70)
Therapeutic hypothermia	115 (6.6)	28 (0.2)	38 (0.1)	42 (0.02)	>100	13.83 (8.43-22.69)	2.92 (1.89-4.52)
Mechanical ventilation	73 (4.2)	54 (0.5)	125 (0.2)	280 (0.1)	38.69 (29.93-50.01)	4.30 (3.22-5.75)	1.61 (1.31-2.00)
iNO treatment	14 (0.8)	11 (0.1)	27 (0.04)	40 (0.02)	45.98 (24.94-84.74)	5.27 (2.68-10.36)	2.31 (1.39-3.81)
Seizures	61 (3.5)	43 (0.4)	136 (0.2)	278 (0.1)	32.88 (24.91-43.39)	3.33 (2.41-4.60)	1.70 (1.38-2.08)
Low Apgar score							
5-min <4	101 (5.8)	68 (0.6)	130 (0.2)	227 (0.1)	63.88 (51.60-80.63)	6.28 (4.79-8.25)	2.00 (1.61-2.49)
5-min <7	290 (16.6)	330 (2.8)	534 (0.7)	816 (0.3)	50.00 (44.00-56.84)	8.27 (7.28-9.41)	2.24 (2.01-2.50)
Respiratory							
CPAP	717 (41.1)	1976 (16.6)	4286 (5.9)	7442 (2.9)	13.74 (12.88-14.66)	5.55 (5.30-5.82)	1.97 (1.90-2.05)
Meconium aspiration	73 (4.2)	150 (1.3)	359 (0.5)	610 (0.2)	15.82 (12.42-20.16)	4.67 (3.89-5.60)	1.87 (1.64-2.14)
Hypoglycemia	374 (21.5)	2449 (20.6)	4914 (6.7)	9993 (3.9)	5.46 (4.90-6.08)	5.27 (5.03-5.51)	1.76 (1.70-1.82)

Abbreviations: ARR, adjusted risk ratio; CPAP, continuous positive airway pressure; iNO, treatment with inhaled nitric oxide; UC-pH, umbilical cord pH.

^a^ Calculated as the ratio between outcomes in the groups compared with outcomes in the group with UC-pH levels of 7.20 to 7.50 (reference group). Results were adjusted for infant sex assigned at birth, gestational age, year of birth, intrapartum fever, birth weight less than −2 SDs, and type 1 or 2 diabetes.

^b^ Includes neonatal death, therapeutic hypothermia, mechanical ventilation, treatment with inhaled nitric oxide, or seizures.

In the sensitivity analyses, we found that restricting the composite outcome to exclude therapeutic hypothermia resulted in a reduced risk in the group with UC-pH levels of less than 7.00 (Table 3). Results from the analysis with a 7-day study period and the unadjusted results were not markedly different from the main results (eTables 2 and 3 in Supplement 1). When only including cases with UC-pH levels measured from both the umbilical cord artery and vein, we found a lower risk of adverse outcomes in the group with UC-pH levels of less than 7.00, but nearly the same risk in the groups with UC-pH levels of 7.00 to 7.09 and 7.10 to 7.19 (eTable 4 in Supplement 1).

**Table 3.** Comparison of Different Composite Outcomes Between UC-pH Groups

Outcome	UC-pH group, No. (%)				ARR (95% CI)^a^		
	<7.00 (n = 1743)	7.00-7.09 (n = 11 904)	7.10-7.19 (n = 73 244)	7.20-7.50 (n = 253 540)	<7.00	7.00-7.09	7.10-7.19
Composite, 0-28 d							
Neonatal death, therapeutic hypothermia, mechanical ventilation, iNO treatment, or seizures	171 (9.8)	101 (0.8)	259 (0.4)	576 (0.2)	43.93 (37.19-51.90)	3.83 (3.10-4.74)	1.60 (1.38-1.85)
Neonatal death, mechanical ventilation, iNO treatment, or seizures	121 (6.9)	91 (0.8)	248 (0.3)	563 (0.2)	31.88 (26.26-38.71)	3.57 (2.86-4.46)	1.57 (1.35-1.82)
Neonatal death, mechanical ventilation, or seizures	120 (6.9)	91 (0.8)	246 (0.3)	558 (0.2)	31.90 (26.30-38.69)	3.55 (2.84-4.43)	1.57 (1.35-1.82)
Composite, 0-7 d							
Neonatal death, therapeutic hypothermia, mechanical ventilation, iNO treatment, or seizures	171 (9.8)	100 (0.8)	241 (0.3)	490 (0.2)	51.74 (43.62-61.35)	4.44 (3.58-5.52)	1.74 (1.49-2.03)
Neonatal death, mechanical ventilation, iNO treatment, or seizures	121 (6.9)	89 (0.7)	230 (0.3)	477 (0.2)	37.42 (30.70-45.60)	4.10 (3.27-5.15)	1.72 (1.47-2.01)
Neonatal death, mechanical ventilation, or seizures	120 (6.9)	89 (0.7)	229 (0.3)	474 (0.2)	37.51 (30.80-45.67)	4.08 (3.26-5.13)	1.71 (1.46-2.00)

Abbreviations: ARR, adjusted risk ratio; iNO, treatment with inhaled nitric oxide; UC-pH, umbilical cord pH.

^a^ Calculated as the ratio between outcomes in the groups compared with outcomes in the group with UC-pH levels of 7.20 to 7.50 (reference group). Results were adjusted for infant sex assigned at birth, gestational age, year of birth, intrapartum fever, birth weight less than −2 SDs, and type 1 or 2 diabetes.

In the group with missing UC-pH measurements (31 285 of 362 389 [5.9%] of the study population), we found similar characteristics as in the groups with UC-pH levels of greater than 7.10 and similar risk of adverse outcomes as in the group with UC-pH levels of 7.10 to 7.19, except for the risk of neonatal death, which equaled that of the group with UC-pH levels of 7.00 to 7.09 (eTables 5 and 6 in Supplement 1). Multiple imputation for missing UC-pH measurement did not change the overall results (eTable 7 in Supplement 1).

## Discussion

In this nationwide cohort study, we investigated the risk of neonatal morbidity and mortality in infants with low UC-pH levels and found that neonates with lower UC-pH values had substantially increased risk of adverse neonatal outcomes. This was seen in the group with UC-pH levels of less than 7.00 but also in the group with UC-pH levels of 7.00 to 7.09 and, to a lesser extent, in the group with UC-pH levels of 7.10 to 7.19. Our findings indicate an association between UC-pH levels and the risk of neonatal morbidity and mortality, even at levels not previously clearly related to adverse neonatal outcomes.

Our results correlate well with those of previous studies indicating that the risk of an adverse outcome is increased at UC-pH levels above 7.00. Our results showed that the ARR for therapeutic hypothermia was 13.83 (95% CI, 8.43-22.69) in the group with UC-pH levels of 7.00 to 7.09 despite the pH threshold at 7.00 recommended in the Danish guidelines (Table 2).^29^ This supports previous findings suggesting a higher pH threshold for screening for HIE and using therapeutic hypothermia if other criteria are fulfilled.^7,8,9,10^ In line with Bligard et al,^11^ who found a tripled risk of respiratory distress if UC-pH value was less than 7.20, we found that the ARR for continuous positive airway pressure to support transition to extrauterine life was 5.55 (95% CI, 5.30-5.82) in the group with UC-pH of 7.00 to 7.09 and 1.97 (95% CI, 1.90-2.05) in the group with UC-pH levels of 7.10 to 7.19.

It is known from previous studies^30,31,32^ that there is an association among acidosis, hypoglycemia and adverse neurological outcomes. However, we have not found prior studies indicating similar marked (5-fold) increased risk of hypoglycemia in infants with UC-pH levels of below 7.10.

The correlation between fetal acidosis and adverse neonatal outcome is complex and not well understood. Adverse neonatal outcomes can be caused by intrapartum acidosis alone or by known or unknown antenatal conditions such as maternal medical diseases, late gestational age, placental insufficiency, infections, fetal anemia, acute obstetric situations such as placental abruption, or a combination of acidosis and 1 or more of these factors.^19,20,21,22,23,33,34^ This means that in individual cases, differentiating whether neonatal encephalopathy is primarily caused by hypoxia (eg, HIE), inherent or acquired child frailty, birth-related factors, or a combination thereof, can be challenging or even impossible.^34,35,36^ The complexity implies difficulty in establishing a model linking umbilical cord acidemia with a risk of neonatal morbidity and mortality without introducing bias. In our main analysis, we adjusted for factors known from the literature to affect the risks of hypoxia and adverse neonatal outcomes.

Most previous studies^9,10,11,12^ restricted the inclusion criteria to infants with UC-pH measured from both the umbilical cord artery and vein. This criterion ensures that the pH level from the umbilical artery, which defines acidosis, is known. However, previous studies^13^ have shown that the registration of only 1 UC-pH measurement is higher in cases with increased risk of complications (eg, fetal growth retardation), and excluding these infants may therefore underestimate the risk of complications. In the present study we expanded the inclusion criteria to infants with at least 1 UC-pH measurement. When including infants with 1 UC-pH measurement (or 2 measurements with a pH difference <0.02, suggesting sampling from the same vessel), part of our results were expectedly based on UC-pH measurement from the umbilical vein. Samples obtained from the umbilical vein have a higher pH level, and this might cause selective misclassification in the UC-pH groups. Therefore, the group with UC-pH levels of less than 7.00 is potentially slightly underestimated, and some of the infants in the group with UC-pH levels of 7.00 to 7.09 will expectedly include infants with an arterial pH level below 7.00. To compare our results with those from previous studies, we performed a sensitivity analysis in which only infants with UC-pH levels sampled from both the umbilical artery and vein were included. This analysis showed a lower risk of adverse outcomes in the group with UC-pH levels of less than 7.00, but the risk of adverse outcomes in the groups with UC-pH levels of 7.00 to 7.09 and 7.10 to 7.19 were the same. This corresponds well with a previous study showing that in very acute situations with expected higher risk of adverse outcomes, the proportion with only 1 UC-pH measurement was higher.^13^ Despite the risk of misclassification, it is valuable to include infants with only 1 UC-pH measurement to include the most serious cases.

### Strengths and Limitations

The strength of our study lies in the national population-based material that makes it possible to study the risk of adverse neonatal outcome according to UC-pH levels while avoiding selection bias from a selective approach only measuring UC-pH levels in infants suspected of having or with an increased risk of hypoxia and neonatal complications. The large sample size allowed us to include rare events such as therapeutic hypothermia and neonatal death. The national implementation of universal UC-pH measurement means that the cohort included deliveries and infants from all parts of the country and from both small maternity wards and large university hospitals. This is unique compared with previous studies,^9,10,12,33^ which are from single hospitals or administrative regions. The comprehensive registration of characteristics, interventions, and complications during birth enabled us to compare the 4 UC-pH level groups and the group with missing UC-pH measurement according to risk factors for acidosis and complications.

This study also has some limitations. Our study did not contain all elements of the cord blood gas analyses, since only UC-pH measurements are included in the central registers in Denmark. Not having access to Pco_2_, base excess, and lactate values means that we were not able to distinguish between metabolic and respiratory acidosis. However, previous studies^7,37,38^ have shown that the arterial UC-pH measurement had a markedly better sensitivity to detect moderate to severe HIE compared with umbilical cord base excess, and UC-pH levels in general are recognized as a good parameter of adverse neonatal outcomes.

The proportion of infants with missing UC-pH values within the composite outcome was 109 of 1216 (9.0%) and within neonatal death was 27 of 141 (19.1%), which is in contrast to 5.9% missing UC-pH values in the overall population. This could have resulted in an underestimation of the risk of adverse outcomes in the groups with low UC-pH levels. However, when testing with multiple imputations for missing values, we found that effect estimates were largely unchanged.

Another limitation is that the validity of the study depends on registrations of diagnoses and procedures by clinicians, which will never be flawless in everyday clinical life. In general, registrations of treatments and interventions are more valid than diagnoses.^39^ We found no indications of differences in registration between different UC-pH groups.

The primary composite outcome was, in addition to neonatal death, based on procedures used in the treatment of severe acidemia or respiratory diseases. We acknowledge that including therapeutic hypothermia in the composite primary outcome affects the results, since UC-pH levels of less than 7.00 constitute one of the criteria for this treatment in Danish guidelines. This was also seen from the sensitivity analysis with restricted composite outcome (Table 3).

Our study was conducted in a high-income country where more than 95% of deliveries take place in a hospital and where there is good access to ultrasonographic examinations for due dates and screening for malformations as well as obstetric and neonatal care. This may limit the generalizability of the results if compared with other settings. The risk of adverse neonatal outcomes associated with acidosis is presumably higher in settings with less access to obstetric and neonatal care.

## Conclusions

This comprehensive cohort study with universal UC-pH measurement indicates that a UC-pH level below 7.20 is associated with a higher risk of infant mortality and morbidities. Even UC-pH levels of 7.10 to 7.19 were linked to an increased risk of severe neonatal morbidities, and neonatal morbidity increased further when UC-pH levels were below 7.10. Furthermore, the results indicated that UC-pH levels of 7.00 to 7.10 were associated with a 5-fold increased need for respiratory support and risk of hypoglycemia. Although we could not differentiate between the specific adverse effects of hypoxia from birth and other causes of neonatal complications or interventions, recognizing the risks associated with low UC-pH levels remains important. Knowing the risk of neonatal adverse outcomes at different levels of UC-pH may lead to reconsidering the threshold for more intensive observation of infants to identify clinical conditions in which intervention is needed. Future studies are needed, especially concerning the risk of adverse outcomes later in childhood.

## Notes

Supplement 1. **eTable 1**. *ICD-10* Codes **eTable 2.** Comparison of Primary and Secondary Outcomes Between Umbilical Cord pH Groups, 0 to 7 Days After Birth **eTable 3.** Comparison of Primary and Secondary Outcomes Between UC-pH Groups, Unadjusted Results **eTable 4.** Comparison of Primary and Secondary Outcomes Between Umbilical Cord pH Groups in Cohort With 2 Umbilical Cord pH Measurements **eTable 5.** Baseline Descriptive Characteristics According to Umbilical Cord pH Levels, Including Missing Umbilical Cord pH Measurements **eTable 6.** Comparison of Primary and Secondary Outcomes Among Infants With Missing Umbilical Cord pH Measurements and Reference Group **eTable 7.** Comparison of Primary and Secondary Outcomes Between Umbilical Cord pH Groups With Imputation for Missing Values of Umbilical Cord pH

Supplement 2.
Data Sharing Statement
