# Supplementary material for: Umbilical Cord pH Levels and Neonatal Morbidity and Mortality
Source: JAMA Netw Open. 2024 Aug 14;7(8):e2427604. doi: 10.1001/jamanetworkopen.2024.27604 (PMC11325209; doi:10.1001/jamanetworkopen.2024.27604)
Supplement: Supplement 1. — eTable 1. ICD-10 Codes eTable 2. Comparison of Primary and Secondary Outcomes Between Umbilical Cord pH Groups, 0 to 7 Days After Birth eTable 3. Comparison of Primary and Secondary Outcomes Between UC-pH Groups, Unadjusted Results eTable 4. Comparison of Primary and Secondary Outcomes Between Umbilical Cord pH Groups in Cohort With 2 Umbilical Cord pH Measurements eTable 5. Baseline Descriptive Characteristics According to Umbilical Cord pH Levels, Including Missing Umbilical Cord pH Measurements eTable 6. Comparison of Primary and Secondary Outcomes Among Infants With Missing Umbilical Cord pH Measurements and Reference Group eTable 7. Comparison of Primary and Secondary Outcomes Between Umbilical Cord pH Groups With Imputation for Missing Values of Umbilical Cord pH [file jamanetwopen-e2427604-s001.pdf]

Supplementary Online Content

Andersson CB, Klingenberg C, Thellesen L, Johnsen SP, Kesmodel UK, Petersen JP. Umbilical cord pH levels and neonatal morbidity and mortality. *JAMA Netw Open*. 2024;7(8):e2427604. doi:10.1001/jamanetworkopen.2024.27604

eTable 1. *ICD-10* Codes

eTable 2. Comparison of Primary and Secondary Outcomes Between Umbilical Cord pH Groups, 0 to 7 Days After Birth

eTable 3. Comparison of Primary and Secondary Outcomes Between Umbilical Cord pH Groups, Unadjusted Results

eTable 4. Comparison of Primary and Secondary Outcomes Between Umbilical Cord pH Groups in Cohort With 2 Umbilical Cord pH Measurements

eTable 5. Baseline Descriptive Characteristics According to Umbilical Cord pH Levels, Including Missing Umbilical Cord pH Measurements

eTable 6. Comparison of Primary and Secondary Outcomes Among Infants With Missing Umbilical Cord pH Measurements and Reference Group

eTable 7. Comparison of Primary and Secondary Outcomes Between Umbilical Cord pH Groups With Imputation for Missing Values of Umbilical Cord pH

This supplementary material has been provided by the authors to give readers additional information about their work.

© 2024 Andersson CB et al. *JAMA Network Open*.

eTable 1. *ICD10* Codes

	ICD 10 Codes
**Infant**	
CPAP	BGFC32
Mechanical ventilation	BGDA0
Meconium aspiration	P24
iNO treatment	BGXA71
Hypoglycemia	Q2 Q30 Q31 Q32 Q33 Q34 Q0
Seizures	P90
Therapeutic hypothermia	BMFL38B
**Pregnancy**	
Smoking in pregnancy	Z35M18 UT2
Type 1 or 2 diabetes	O240 O241 O242 O245 O249 E1
Gestational diabetes	O244
Hypertension	O10 O13 O16 I1
Preeclampsia	O11 O14 O15
Other medical diseases	O266G O990 O992B E03 O992C E05 O993A G35 G40 O995 J44 J45 Z980 O996A K50 O996B K51 M05 M06 O6 M32 R768D DE282
Placental insufficiency	O363 O365 O368E O368F O410
**Birth**	
Intrapartum fever	O750
Serious births events	O710 O711 O690 O660 O431E
Breech (vaginal delivery)	O641 UP07 UP08 UP09 UP10 UP11 UP19
Instrumental delivery	KMAE00 KMAE03 KMAE20 KMAE96 KMAF00 KMAF10 KMAF20 KMAF96
Emergency CS	KMCA10A KMCA10E
Hypoglycemia	Q2 Q30 Q31 Q32 Q33 Q34 Q0
Seizures	P90
Therapeutic hypothermia	BMFL38B

Abbreviations: CPAP, continuous positive airway pressure; ; iNO, treatment with inhaled Nitric oxide, CS, Cesarean Section

© 2024 Andersson CB et al. *JAMA Network Open*.

eTable 2. Comparison of Primary and Secondary Outcomes Between Umbilical Cord pH Groups, 0-7 Days after Birth

	**Umbilical cord pH[a] No. (%) if infants**				**Adjusted risk ratios (95% CI) [b]**		
	**<7.00**	**7.00-7.09**	**7.10-7.19**	**7.20-7.50**	**<7.00 vs. 7.20-7.50**	**7.00-7.09 vs. 7.20-7.50**	**7.10-7.19 vs. 7.20-7.50**
**Primary composite outcome[c]**	171 (9.8)	100 (0.8)	241 (0.3)	490 (0.2)	51.74 (43.62-61.35)	4.44 (3.58-5.52)	1.74 (1.49-2.03)
**Secondary outcomes**							
**Individual components of the primary outcome**							
Neonatal death	31 (1.8)	9 (0.1)	13 (0.0)	33 (0.0)	>100	6.41 (3.03-13.59)	1.49 (0.79-2.80)
Therapeutic hypothermia	116 (6.7)	29 (0.2)	39 (0.1)	42 (0.0)	>100	14.31 (8.78-23.35)	2.98 (1.93-4.60)
Mechanical ventilation	73 (4.2)	53 (0.4)	121 (0.2)	243 (0.1)	44.67 (34.40-58.00)	4.88 (3.63-6.56)	1.80 (1.45-2.24)
iNO treatment	14 (0.8)	11 (0.1)	28 (0.0)	40 (0.0)	45.61 (24.72-84.13)	5.27 (2.68-10.37)	2.37 (1.45-3.90)
Seizures	61 (3.5)	41 (0.3)	121 (0.2)	236 (0.1)	38.52 (29.02-51.13)	3.71 (2.65-5.18)	1.77 (1.42-2.20)
**Low Apgar scores**							
Five-minute Apgar score < 4	101 (5.8)	68 (0.6)	130 (0.2)	227 (0.1)	63.90 (50.62-80.66)	6.28 (4.79-8.25)	2.00 (1.61-2.48)
Five-minute Apgar score < 7	291 (16.7)	330 (2.8)	534 (0.7)	817 (0.3)	50.12 (44.10-57.00)	8.26 (7.27-9.40)	2.23 (2.00-2.50)
**Respiratory outcomes**							
CPAP	715 (41.0)	1975 (16.6)	4201 (5.7)	7084 (2.8)	14.33 (13.42-15.29)	5.80 (5.53-6.09)	2.03 (1.95--2.10)
Meconium aspiration	72 (4.1)	147 (1.2)	341 (0.5)	527 (0.2)	17.65 (13.79-22.60)	5.17 (4.28-6.24)	2.02 (1.76-2.32)
**Hypoglycemia**	374 (21.4)	2450 (20.6)	4923 (6.7)	10 010 (3.9)	5.41 (4.85-6.02)	5.26 (5.03-5.51)	1.76 (1.70-1.82)

Abbreviations: RR, risk ratio; CPAP, continuous positive airway pressure; ; iNO, treatment with inhaled Nitric oxide.
[a] Data are presented as the number (percentage) of infants.
[b] Adjusted risk ratios were calculated as the ratio between outcomes in the groups compared to outcomes in the group with Umbilical cord pH levels 7.20-7.50. Results were adjusted for infant sex assigned at birth; GA; year of birth; intrapartum fever; birth weight < -2 SD, and type I or 2 diabetes
[c] Primary composite outcome: Neonatal death (0-7 days), therapeutic hypothermia, mechanical ventilation, treatment with inhaled Nitric oxide (iNO) or seizures.
PH <7.0: n=1743, pH 7.00-7.09: n= 11 904, pH 7.10-7.19: n= 73 244, pH 7.20-7.50: n= 253 540

© 2024 Andersson CB et al. *JAMA Network Open*.

eTable 3. Comparison of Primary and Secondary Outcomes Between Umbilical Cord pH Groups, Unadjusted Results

Outcomes	Unadjusted risk ratios (95% CI)[a]		
	**<7.00 vs. 7.20-7.50**	**7.00-7.09 vs. 7.20-7.50**	**7.10-7.19 vs. 7.20-7.50**
**Primary composite outcome[b]**	43.18 (36.65-50.88)	3.74 (3.03-4.61)	1.56 (1.35-1.80)
**Secondary outcomes**			
**Individual components of the primary outcome**			
Neonatal death	89.92 (58.78- >100)	4.26 (2.23-8.14)	0.88 (0.49-1.58)
Therapeutic hypothermia	>100	14.20 (8.81-22.90)	3.13 (2.02-4.86)
Mechanical ventilation	37.92 (29.44-48.85)	4.11 (3.07-5.49)	1.55 (1.25-1.91)
iNO treatment	50.91 (27.75-93.40)	5.86 (3.01-11.41)	2.34 (1.43-3.81)
Seizures	31.92 (24.29-41.94)	3.29 (2.39-4.54)	1.69 (1.38-2.08)
**Low Apgar scores**			
Five-minute Apgar score <4	64.72 (51.44-81.44)	6.38 (4.87-8.36)	1.98 (1.60-2.46)
Five-minute Apgar score <7	51.69 (45.60-58.60)	8.61 (7.59-9.78)	2.27 (2.03-2.53)
**Respiratory outcomes**			
CPAP	14.01 (13.19-14.89)	5.66 (5.40-5.92)	1.99 (1.92-2.07)
Meconium aspiration	17.41 (13.72-22.09)	5.24 (4.39-6.26)	2.0 (1.79-2.32)
**Hypoglycemia**	5.44 (4.96-5.97)	5.22 (5.01-5.43)	1.70 (1.65-1.76)

Abbreviations: RR, risk ratio; CPAP, continuous positive airway pressure; ; iNO, treatment with inhaled Nitric oxide.
[a] Risk ratios were calculated as the ratio between outcomes in the groups compared to outcomes in the group with Umbilical Cord pH levels 7.20-7.50.
[b] Primary composite outcome: Neonatal death, therapeutic hypothermia, mechanical ventilation, treatment with inhaled Nitric oxide (iNO) or seizures.

© 2024 Andersson CB et al. *JAMA Network Open*.

eTable 4. Comparison of Primary and Secondary Outcomes Between Umbilical Cord pH groups in Cohorts with two Umbilical Cord pH Measurements

Outcomes	Umbilical cord pH No. (%) if infants				Adjusted risk ratios (95% CI)[a]		
	<7.00	7.00-7.09	7.10-7.19	7.20-7.50	<7.00 vs. 7.20-7.50	7.00-7.09 vs. 7.20-7.50	7.10-7.19 vs. 7.20-7.50
**Primary composite outcome[b]**	137 (8.7)	90 (0.8)	233 (0.3)	443 (0.2)	40.97 (33.96-49.44)	3.81 (3.03-4.78)	1.57 (1.34-1.84)
**Secondary outcomes**							
**Individual components of the primary outcome**							
Neonatal death (0-7 days)	25 (1.6)	9 (0.1)	12 (0.0)	44 (0.0)	76.15 (46.18->100)	3.98 (1.93-8.20)	0.85 (0.45-1.60)
Therapeutic hypothermia	91 (5.8)	25 (0.2)	33 (0.1)	25 (0.0)	>100	17.22 (9.62-30.82)	3.52 (2.09-5.93)
Mechanical ventilation	61 (3.7)	49 (0.4)	114 (0.2)	215 (0.1)	35.36 (26.41-47.33)	4.36 (3.20-5.95)	1.61 (1.29-2.02)
iNO treatment	9 (0.6)	9 (0.1)	25 (0.0)	29 (0.0)	35.38 (16.68-75.04)	5.03 (2.32-10.89)	2.45 (1.41-4.24)
Seizures	46 (2.9)	38 (0.3)	125 (0.2)	215 (0.1)	28.72 (20.86-39.54)	3.27 (2.31-4.63)	1.70 (1.36-2.11)
**Low Apgar scores**							
Five-minute Apgar score < 4	82 (5.2)	62 (0.6)	116 (0 .2)	174 (0.1)	58.95 (45.29-76.73)	6.22 (4.65-8.33)	1.93 (1.52-2.45)
Five-minute Apgar score < 7	246 (15.7)	299 (2.7)	478 (0.7)	584 (0.3)	51.91 (44.95-59.95)	8.79 (7.64-10.11)	2.35 (2.08-2.65)
**Respiratory outcomes**							
CPAP	634 (40.4)	1832 (16.4)	3955 (5.7)	5821 (2.9)	13.65 (12.73-14.64)	5.55 (5.28-5.84)	1.95 (1.87-2.03)
Meconium aspiration	62 (34.0)	140 (1.3)	328 (0.5)	496 (0.3)	14.44 (11.08-18.81)	4.51 (3.72-5.46)	1.75 (1.52-2.01)
**Hypoglycemia**	349 (22.2)	2316 (20.)	4536 (6.6)	7786 (3.9)	5.76 (5.15-6.45)	5.37 (5.12-5.64)	1.74 (1.68-1.80)

Abbreviations: RR, risk ratio; CPAP, continuous positive airway pressure; iNO, treatment with inhaled Nitric oxide.
N=282 205
[a] Adjusted risk ratios were calculated as the ratio between outcomes in the groups compared to outcomes in the group with Umbilical Cord pH 7.20-7.50. Results were adjusted for infant sex assigned at birth; GA; year of birth; intrapartum fever; birth weight < -2 SD, and type 1 or 2 diabetes.
[b] Primary composite outcome: Neonatal death, therapeutic hypothermia, mechanical ventilation, treatment with inhaled Nitric oxide (iNO) or seizures.
PH <7.0: n=1571, pH 7.00-7.09: n= 11 184, pH 7.10-7.19: n= 69 256, pH 7.20-7.50: n= 200 194

© 2024 Andersson CB et al. *JAMA Network Open*.

eTable 5. Baseline Descriptive Characteristics According to Umbilical Cord pH levels, Including Missing Umbilical Cord pH Measurements

**Characteristics**	**Umbilical cord pH[a]**				
	**<7.00 (n=1743)**	**7.00-7.09 (n=11 904)**	**7.10-7.19 (n=73 244)**	**7.20-7.50 (n= 253 540)**	**Missing (n=21 385)**
**Infant**					
Gestational age in weeks. Mean, (SD)	40.2 (1.2)	40.2 (1.2)	40.1 (1.1)	39.9 (1.2)	39.9 (1.2)
• 37	82 (4.7)	430 (3.6)	2727 (3.7)	12 648 (5.0)	1057 (4.9)
• 38	162 (9.3)	1071 (9.0)	7496 (10.2)	37 951 (15.0)	2835 (13.3)
• 39	318 (19.1)	2229 (18.7)	14 819 (20.2)	61 688 (24.3)	5198 (24.3)
• 40	568 (32.6)	3763 (31.6)	24 028 (32.8	76 299 (30.1)	6882 (32.3)
• 41	555 (31.8)	4000 (33.6)	22 041 (30.1)	60 045 (23.7)	5028 (23.5)
• ≥42	58 (3.5)	411 (3.5)	2133 (3.0)	4909 (1.9)	385 (1.8)
Sex assigned at birth, male	1020 (55.4)	6522 (54.8)	39 468 (53.9)	127 602 (50.3)	9565 (44.7)
Sex assigned at birth, female	777 (44.6)	5382 (45.2)	33 776 (46.1)	125 938 (49.7)	9812 (45.9)
Birthweight in grams. Mean, SD	3552 (537)	3607 (491)	3615 (477)	3545 (478)	3534 (484)
• SGA <1 SD	265 (15.2)	1469 (12.3)	7645 (10.4)	27 525 (10.9)	2276 (10.6)
• SGA <2 SD	78 (4.5)	1469 (12.3)	2200 (3.0)	27 525 (10.9)	703 (3.3)
• LGA >2 SD	150 (8.6)	1216 (10.2)	7729 (10.6)	24267 (9.6)	1800 (8.4)
**Pregnancy**					
Smoking in pregnancy	117 (6.7)	668 (5 .6)	4621 (6.3)	18 796 (7.4)	1551 (7.3)
Type 1 or 2 diabetes	15 (0.9)	115 (1.0)	659 (0.9)	1932 (0.8)	124 (0.6)
Gestational diabetes	75 (4.3)	492 (4.1)	2960 (4.0)	9354 (3.7)	649 (3.0)
Hypertension	49 (2.8)	388 (3.3)	2208 (3.0)	6805 (2.7)	447 (2.1)
Other medical diseases[b]	239 (13.7)	1179 (9.9)	7167 (9.8)	24 342 (9.6)	539 (2.5)
Placental insufficiency[c]	126 (7.2)	544 (4.6)	3394 (4.6)	14 212 (5.6)	1975 (9.2)
**Birth**					
Intrapartum fever	64 (3.7)	578 (4.9)	2915 (4.0)	6275 (2.5)	442 (2.1)
Serious birth events[d]	159 (9.1)	437 (3.7)	1724 (2.4)	3702 (1.5)	304 (1.4)
Breech, vaginal delivery	66 (3.8)	193 (1.6)	882 (1.2)	9038 (3.6)	652 (3.1)
Instrumental delivery	500 (28.9)	2708 (22.8)	9815 (13.4)	12 113 (4.8)	1093 (5.1)
Emergency CS	478 (27.4)	1189 (10.0)	4531 (6.2)	26 814 (10.6)	1681 (7.9)

Abbreviations: SGA, small for gestational age; LGA, large for gestational age; CS, Cesarean section
[a] Data are presented as the number (percentage) of infants unless otherwise indicated.
[b]Other medical diseases: respiratory diseases, hypothyroidism, hyperthyroidism, polycystic ovary syndrome, gastrointestinal diseases, neurological disease, anemia, intrahepatic cholestasis of pregnancy.
[c] Placental insufficiency; signs of fetal distress before delivery; pathological signs on the cardiotocography, intrauterine growth restriction (< - 2 SD), low amnion fluid volumen or abnormal ultrasonographic Doppler flow indexes of fetal vessels.
[d] Serious birth events: Shoulder dystocia, uterine rupture, abruptio placentae, cord prolapse or vasa previa
Birthweight data not available or valid for all infants (2575 missing)

© 2024 Andersson CB et al. *JAMA Network Open*.

eTable 6. Comparison of Primary and Secondary Outcomes Among Infants With Missing Umbilical Cord pH Measurements and Reference Group

	**Missing Umbilical Cord pH No. (%) if infants (n=21 385)**	**Adjusted risk ratios (95% CI)[a] Missing vs. 7.20-7.50**
**Primary outcome[b]**	109 (0.5)	2.27 (1.85-2.78)
**Secondary outcomes**		
**Individual components of the primary outcome**		
Neonatal death	27 (0.1)	5.75 (3.60-9.20)
Therapeutic hypothermia	25 (0.1)	7.23 (4.40-11.89)
Mechanical ventilation	50 (0.2)	2.16 (1.60-2.93)
iNO treatment	8 (0.0)	2.48 (1.15-5.35)
Seizures	44 (0.2)	1.88 (1.37-2.59)
**Low Apgar scores**		
Five-minute Apgar score <4	56 (0.3)	2.94 (2.20-3.95)
Five-minute Apgar score <7	147 (0.7)	2.20 (1.84-2.62)
**Respiratory outcomes**		
CPAP	698 (3.3)	1.17 (1.09-1.27)
Meconium aspiration	54 (0.3)	1.10 (0.80-1.40)
**Hypoglycemia**	830 (3.9)	1.02 (0.95-1.09)

Abbreviations: RR, risk ratio; CPAP, continuous positive airway pressure; ; iNO, treatment with inhaled Nitric oxide.
[a] Adjusted risk ratios were calculated as the ratio between outcomes in the groups compared to outcomes in the group with Umbilical Cord pH levels 7.20-7.50. Results were adjusted for infant sex assigned at birth; GA; year of birth; intrapartum fever; birth weight < -2 SD, and type 1 or 2 diabetes.
[b] Primary composite outcome: Neonatal death, therapeutic hypothermia, mechanical ventilation, treatment with inhaled Nitric oxide (iNO) or seizures.

© 2024 Andersson CB et al. *JAMA Network Open*.

eTable 7. Comparison of Primary and Secondary Outcomes Between Umbilical Cord pH Groups With Imputation for Missing Values of Umbilical Cord pH

	**Adjusted risk ratios (95% CI)[a]**		
	**<7.00 vs. 7.20-7.50**	**7.00-7.09 vs. 7.20-7.50**	**7.10-7.19 vs. 7.20-7.50**
**Primary outcome[b]**	43.95 (37.20-51.92)	3.83 (3.10-4.74)	1.60 (1.38-1.85)
**Secondary outcomes**			
**Individual components of the primary outcome**			
Neonatal death	93.77 (60.50->100)	4.59 (2.38-8.86)	0.95 (0.53-1.69)
Therapeutic hypothermia	>100	13.83 (8.43-22.69)	2.92 (1.89-4.52)
Mechanical ventilation	38.70 (29.94-50.03)	4.30 (3.22-5.75)	1.61 (1.31-1.99)
iNO treatment	44.99 (24.95-84.77)	5.26 (2.68-10.35)	2.31 (1.39-3.81)
Seizures	32.89 (24.92-43.41)	3.33 (2.41-4.59)	1.70 (1.38-2.08)
**Low Apgar scores**			
Five-minute Apgar score <4	63.91 (50.63-80.67)	6.28 (4.79-8.25)	2.00 (1.61-2.49)
Five-minute Apgar score <7	50.02 (44.01-56.86)	8.27 (7.28-9.40)	2.24 (2.00-2.50)
**Respiratory outcomes**			
CPAP	13.75 (12.89-14.66)	5.55 (5.29-5.82)	1.97 (1.90-2.05)
Meconium aspiration	15.81 (12.41-20.14)	4.67 (3.89-5.60)	1.87 (1.64-2.13)
**Hypoglycemia**	5.46 (4.90-6.08)	5.27 (5.03-5.52)	1.76 (1.70-1.81)

Abbreviations: RR, risk ratio; CPAP, continuous positive airway pressure; ; iNO, treatment with inhaled Nitric oxide.
[a] Adjusted risk ratios were calculated as the ratio between outcomes in the groups compared to outcomes in the group with Umbilical Cord pH levels 7.20-7.50. Results were adjusted for infant sex assigned at birth; GA; year of birth; intrapartum fever; birth weight < -2 SD, and type 1 or 2 diabetes.
[b] Primary composite outcome: Neonatal death, therapeutic hypothermia, mechanical ventilation, treatment with inhaled Nitric oxide (iNO) or seizures.

© 2024 Andersson CB et al. *JAMA Network Open*.
