# Supplementary material for: Umbilical Cord pH Levels and Neonatal Morbidity and Mortality
Source: JAMA Netw Open. 2024 Aug 14;7(8):e2427604. doi: 10.1001/jamanetworkopen.2024.27604 (PMC11325209; doi:10.1001/jamanetworkopen.2024.27604)
Supplement: Supplement 2. — Data Sharing Statement [file jamanetwopen-e2427604-s002.pdf]

Data Sharing Statement

Andersson. Umbilical Cord pH Levels and Neonatal Morbidity and Mortality. *JAMA Netw Open*. Published August 14, 2024. doi:10.1001/jamanetworkopen.2024.27604

Data
Data available: No

Additional Information
Explanation for why data not available: Data will not be made available for others according to Danish data protection legislation but can be obtained from the Danish registers upon application.
